# An EM algorithm to improve the estimation of the probability of clonal relatedness of pairs of tumors in cancer patients

**DOI:** 10.1186/s12859-019-3148-z

**Published:** 2019-11-08

**Authors:** Audrey Mauguen, Venkatraman E. Seshan, Irina Ostrovnaya, Colin B. Begg

**Affiliations:** Department of Epidemiology and Biostatistics, Memorial Sloan Kettering Cancer Center, 485 Lexington Ave, 2nd floor, New York, NY, 10017 USA

**Keywords:** Cancer, Clonality, EM algorithm, Tumor mutation, Parameter estimation, Random effect model

## Abstract

**Background:**

We previously introduced a random-effects model to analyze a set of patients, each of which has two distinct tumors. The goal is to estimate the proportion of patients for which one of the tumors is a metastasis of the other, i.e. where the tumors are clonally related. Matches of mutations within a tumor pair provide the evidence for clonal relatedness. In this article, using simulations, we compare two estimation approaches that we considered for our model: use of a constrained quasi-Newton algorithm to maximize the likelihood conditional on the random effect, and an Expectation-Maximization algorithm where we further condition the random-effect distribution on the data.

**Results:**

In some specific settings, especially with sparse information, the estimation of the parameter of interest is at the boundary a non-negligible number of times using the first approach, while the EM algorithm gives more satisfactory estimates. This is of considerable importance for our application, since an estimate of either 0 or 1 for the proportion of cases that are clonal leads to individual probabilities being 0 or 1 in settings where the evidence is clearly not sufficient for such definitive probability estimates.

**Conclusions:**

The EM algorithm is a preferable approach for our clonality random-effect model. It is now the method implemented in our R package *Clonality*, making available an easy and fast way to estimate this model on a range of applications.

## Background

Many studies have been published over the past 20 years that involved examining pairs of tumors at the molecular level from a set of patients to determine if, for some patients, the tumors are clonal, i.e. one of the tumors is a metastasis of the other tumor. We focus in this article on the setting where the data comprise somatic mutations from a panel of genes. Various statistical methods have been proposed in the literature. One approach has been to characterize the evidence for clonality using an index of clonal relatedness (see [1] and [2]). However in constructing the index these authors have focused solely on mutations that are shared between the two tumors, ignoring the information from mutations that occur in one tumor but not the other, evidence that argues against clonal relatedness. Other authors have used the proportion of observed mutations that are shared as the index [3, 4], while Bao et al. [5] formalized this idea by assuming that the matched mutations follow a binomial distribution. All of these approaches analyze each case independently. To our knowledge, the approach we discuss in this article, improving upon Mauguen et al. [6], is the only available method that models the data from all cases collectively to obtain parametric estimates of the proportion of cases in the population that are clonal. Also our method relies heavily on the recognition of the fact that the probabilities of occurrence of the observed mutations are crucially informative,especially for shared mutations. Motivated by a study of contralateral breast cancer that will be described in more detail in the next section, we developed a random-effects model to simultaneously analyze each case for clonal relatedness and to obtain an estimate of how frequently this occurs [6]. The corresponding function mutation.rem has been added to the R package *Clonality*, originally described in Ostrovnaya et al. [7]. Overall, the properties of this model were demonstrated to be quite good, in the sense that the parameter estimation has generally low bias except in small samples, *ie* where only a few cases from the population are available [6]. Recently, in applying the model anecdotally, we noticed that in such small datasets, examples can arise where the maximum likelihood estimator of the proportion of clonal cases is zero, even when mutational matches have been observed in some cases. This tends to occur if the absolute number of cases with matches is small, either because the overall number of cases is small, or the proportion of cases that are clonal is small, or in clonal cases the proportion of mutations that are matches is small. This is problematic because it renders the probabilities of clonal relatedness to be exactly zero for all individual cases, an estimate that seems unreasonable, especially if matches on rare mutations have been observed. We thus became interested in alternate estimation methods. In this article we compare estimates obtained by the EM algorithm versus our first approach using a one-step estimate of the conditional likelihood.

## Motivating example

We use data from a study that involved 49 women with presumed contralateral breast cancer [8]. That is, in all of these women the cancers in the opposite breasts were diagnosed clinically as independent primary breast cancers. The tumors were retrieved from the pathology archives at Memorial Sloan Kettering Cancer Center and subjected to sequencing using a panel of 254 genes known or suspected to be important in breast cancer. The key data, i.e. the numbers of mutations and matches for each case, as well as the probability of occurrence for the matched mutations, are reproduced in Table 1. The probabilities of occurrence of each specific mutation are considered known, but must actually be estimated from available sources, such as the Cancer Genome Atlas [9]. Six of the 49 cases had at least 1 mutational match, i.e. exactly the same mutation in both tumors. For 3 of these cases the match was observed at the common *PIK3CA* H1047R locus, known to occur in approximately 14% of all breast cancers. We note that common mutations like this one can vary by disease sub-type but we elect to use probabilities associated with breast cancer overall since the study has a mix of sub-types. Since it is plausible these common mutations could occur by chance in a pair of independent breast cancers, the evidence for clonal relatedness is much less strong than for the other 3 cases with matches at rarely occurring loci, something very unlikely to happen in independent tumors.

**Table 1:** Study of contralateral breast cancers

Case #	Somatic mutations			Details of matches	
	Left breast	Right breast	Matches	Mutations	Probabilities
1	9	7	0		
2	3	3	0		
3	2	7	0		
4	8	10	0		
6	6	5	0		
8	6	2	1	*ARID1A* E250fs	< 1/1000
9	2	3	0		
12	14	3	0		
13	3	3	0		
15	8	5	0		
16	10	8	0		
17	6	8	0		
18	8	2	0		
21	4	3	0		
23	10	4	0		
24	4	3	0		
25	4	6	0		
26	6	5	0		
27	4	5	0		
29	3	1	0		
30	6	5	0		
31	6	5	0		
32	5	4	0		
33	6	4	0		
35	5	4	0		
36	3	4	3	*CDH1* S111fs	< 1/1000
				*TBX3* T267fs	< 1/1000
				*EPPK1* R2337H	< 1/1000
38	8	2	0		
40	10	1	0		
41	0	9	0		
43	4	4	0		
44	9	21	0		
45	3	4	0		
48	2	3	2	*MLH3* M346R	< 1/1000
				*MAP3K1* R248*	< 1/1000
52	5	7	0		
56	2	5	0		
58	3	4	0		
59	2	3	0		
62	4	4	0		
63	3	9	1	*PIK3CA* H1047R	0.137
64	5	4	0		
66	33	3	0		
67	4	1	1	*PIK3CA* H1047R	0.137
70	5	2	0		
71	3	1	0		
72	1	3	0		
74	2	1	0		
75	4	3	1	*PIK3CA* H1047R	0.137
76	7	5	0		
77	3	1	0		

When we apply our random-effects analysis to these data, described in more detail in the “Methods” section, our estimate of the proportion of cases that are clonal (denoted henceforth by *π*) is 0.059, close to the proportion 3/49, reflecting the fact that the model appears to consider the 3 cases with rare matches as clonal and the 3 cases with the common matches as independent. Estimation problems can occur, however, in datasets very similar to this one. For example, when we eliminate from the analysis the two cases that are most clearly clonal, cases *#*36 and *#*48, the estimate of *π* is 0, despite the fact that case *#*8 possesses a very rare match pointing strongly to clonal relatedness. Thus, a different estimation method that reduces the frequency with which boundary estimates of *π* occur is advisable.

## Results

**Fig. 1:**
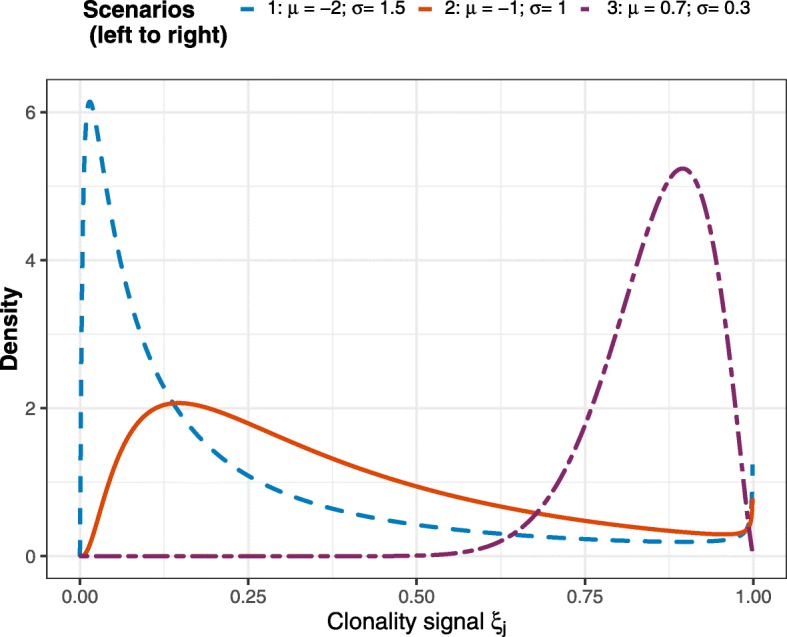
Log-normal distributions of the clonality signal

Simulations were conducted for sample sizes of 25, 50 and 100, with the population proportion of clonal cases (*π*) ranging from 0.10 to 0.75. The distribution of the clonality signal is characterized by 3 different lognormal distributions plotted in Fig. 1. These three scenarios represent, respectively, settings where a small proportion of mutations in a clonal case will be matched (scenario 1), where most of these mutations will be matched (scenario 3), and an intermediate scenario. Note that scenario 1 is particularly problematic for estimation, especially when *π* is small, since in this setting few of the cases will be clonal and these few clonal cases will tend to have few, if any, matches.

Table 2 presents the simulation results for the estimates of *π* averaged over 500 simulations for each setting, along with the standard deviations and ranges of the estimates. Biases can be obtained by comparing these averages with the true value of *π* in the second column of the table. These biases are generally modest, though it is noteworthy that our original one-step approach tends to have positive biases while the approach using the full likelihood and the EM algorithm generally leads to negative bias. More importantly, Table 2 also reports the numbers of times the estimates were exactly on the boundary, i.e. 0 or 1. These occurrences are much less frequent using the EM algorithm and are mostly limited to the small case sample (N =25), low *π* (0.10) setting. The columns on the right-hand side of Table 2 summarize the results using the EM approach for those datasets in which the one-step maximization produced an estimate of *π* of either 0 or 1. These estimates are similar to the true *π*, showing the improved performance with the EM estimation strategy.

**Table 2:** Simulation results

			One-step maximization				EM algorithm				EM algorithm - subset			
N cases	True *π*	Scenario	mean	(sd)	range	N 0-1	mean	(sd)	range	N 0-1	mean	(sd)	range	N 0-1
100	0.10	1: *μ*=−2;*σ*=1.5	0.127	(0.126)	0.010-1.000	0-7	0.086	(0.036)	0.010-0.202	0-0	0.076	(0.037)	0.034-0.138	0-0
		2: *μ*=−1;*σ*=1.0	0.105	(0.038)	0.020-0.234	0-0	0.099	(0.033)	0.020-0.212	0-0				
		3: *μ*=0.7*σ*=0.3	0.101	(0.031)	0.030-0.220	0-0	0.101	(0.031)	0.030-0.220	0-0				
	0.25	1: *μ*=−2;*σ*=1.5	0.259	(0.091)	0.079-0.729	0-0	0.214	(0.051)	0.077-0.387	0-0				
		2: *μ*=−1;*σ*=1.0	0.250	(0.049)	0.121-0.387	0-0	0.245	(0.047)	0.121-0.377	0-0				
		3: *μ*=0.7*σ*=0.3	0.252	(0.043)	0.130-0.380	0-0	0.252	(0.043)	0.130-0.380	0-0				
	0.50	1: *μ*=−2;*σ*=1.5	0.518	(0.113)	0.245-0.881	0-0	0.440	(0.066)	0.230-0.621	0-0				
		2: *μ*=−1;*σ*=1.0	0.498	(0.055)	0.325-0.640	0-0	0.490	(0.054)	0.319-0.624	0-0				
		3: *μ*=0.7*σ*=0.3	0.498	(0.049)	0.350-0.620	0-0	0.498	(0.049)	0.350-0.620	0-0				
	0.75	1: *μ*=−2;*σ*=1.5	0.756	(0.116)	0.495-1.000	0-31	0.662	(0.068)	0.477-0.924	0-0	0.758	(0.052)	0.623-0.924	0-0
		2: *μ*=−1;*σ*=1.0	0.747	(0.050)	0.616-0.881	0-0	0.738	(0.049)	0.609-0.875	0-0				
		3: *μ*=0.7*σ*=0.3	0.748	(0.043)	0.630-0.850	0-0	0.748	(0.043)	0.630-0.850	0-0				
50	0.10	1: *μ*=−2;*σ*=1.5	0.138	(0.193)	0.000-1.000	19-18	0.083	(0.049)	0.000-0.265	11-0	0.083	(0.070)	0.000-0.265	11-0
		2: *μ*=−1;*σ*=1.0	0.113	(0.079)	0.000-1.000	4-1	0.101	(0.048)	0.000-0.272	3-0	0.038	(0.056)	0.000-0.125	3-0
		3: *μ*=0.7*σ*=0.3	0.100	(0.042)	0.000-0.260	2-0	0.100	(0.042)	0.000-0.260	2-0	0	(0.000)	0.000-0.000	2-0
	0.25	1: *μ*=−2;*σ*=1.5	0.270	(0.145)	0.043-1.000	0-4	0.210	(0.071)	0.043-0.456	0-0	0.194	(0.049)	0.122-0.234	0-0
		2: *μ*=−1;*σ*=1.0	0.255	(0.076)	0.100-0.714	0-0	0.245	(0.064)	0.101-0.447	0-0				
		3: *μ*=0.7*σ*=0.3	0.248	(0.061)	0.100-0.440	0-0	0.248	(0.061)	0.100-0.440	0-0				
	0.50	1: *μ*=−2;*σ*=1.5	0.520	(0.154)	0.222-1.000	0-7	0.441	(0.091)	0.212-0.804	0-0	0.64	(0.097)	0.494-0.804	0-0
		2: *μ*=−1;*σ*=1.0	0.501	(0.075)	0.296-0.739	0-0	0.492	(0.073)	0.293-0.713	0-0				
		3: *μ*=0.7*σ*=0.3	0.498	(0.069)	0.320-0.700	0-0	0.498	(0.069)	0.320-0.700	0-0				
	0.75	1: *μ*=−2;*σ*=1.5	0.747	(0.143)	0.480-1.000	0-52	0.659	(0.091)	0.469-0.933	0-0	0.783	(0.072)	0.650-0.933	0-0
		2: *μ*=−1;*σ*=1.0	0.746	(0.075)	0.530-1.000	0-2	0.736	(0.071)	0.527-0.938	0-0	0.926	(0.018)	0.913-0.938	0-0
		3: *μ*=0.7*σ*=0.3	0.746	(0.060)	0.600-0.920	0-0	0.746	(0.060)	0.600-0.920	0-0				
25	0.10	1: *μ*=−2;*σ*=1.5	0.128	(0.197)	0.000-1.000	101-18	0.099	(0.079)	0.000-0.443	46-0	0.088	(0.112)	0.000-0.441	46-0
		2: *μ*=−1;*σ*=1.0	0.118	(0.121)	0.000-1.000	46-4	0.103	(0.063)	0.000-0.365	26-0	0.056	(0.084)	0.000-0.365	26-0
		3: *μ*=0.7*σ*=0.3	0.103	(0.061)	0.000-0.330	29-0	0.101	(0.056)	0.000-0.280	22-0	0.018	(0.045)	0.000-0.228	22-0
	0.25	1: *μ*=−2;*σ*=1.5	0.276	(0.192)	0.000-1.000	6-9	0.216	(0.103)	0.039-0.543	0-0	0.222	(0.108)	0.039-0.432	0-0
		2: *μ*=−1;*σ*=1.0	0.262	(0.110)	0.040-1.000	0-1	0.246	(0.092)	0.040-0.618	0-0	0.176		0.176-0.176	0-0
		3: *μ*=0.7*σ*=0.3	0.250	(0.087)	0.040-0.520	0-0	0.249	(0.088)	0.040-0.520	0-0				
	0.50	1: *μ*=−2;*σ*=1.5	0.515	(0.198)	0.122-1.000	0-19	0.433	(0.124)	0.109-0.878	0-0	0.622	(0.145)	0.384-0.878	0-0
		2: *μ*=−1;*σ*=1.0	0.505	(0.110)	0.201-1.000	0-1	0.492	(0.102)	0.201-0.846	0-0	0.490		0.490-0.490	0-0
		3: *μ*=0.7*σ*=0.3	0.500	(0.096)	0.200-0.760	0-0	0.500	(0.096)	0.200-0.760	0-0				
	0.75	1: *μ*=−2;*σ*=1.5	0.752	(0.175)	0.358-1.000	0-82	0.665	(0.136)	0.332-1.000	0-2	0.835	(0.097)	0.614-1.000	0-2
		2: *μ*=−1;*σ*=1.0	0.752	(0.098)	0.489-1.000	0-5	0.741	(0.094)	0.483-1.000	0-1	0.975	(0.028)	0.941-1.000	0-1
		3: *μ*=0.7*σ*=0.3	0.749	(0.084)	0.480-0.960	0-0	0.749	(0.084)	0.480-0.960	0-0				

The *EM algorithm – subset* results present the estimates obtained with the EM algorithm for the datasets where the one-step maximization gave results on the boundary. *N 0-1* shows the number of times the estimate was exactly 0 - number of times it was exactly 1

The EM approach was used to re-analyze the breast cancer dataset described in the motivating example. When the full dataset of 49 cases is analyzed both methods lead to the same estimate, $\hat {\pi } = 0.059$. However, when cases *#*36 and *#*48 are removed, the EM approach leads to $\hat {\pi } = 0.050$ while the one-step method leads to the boundary value of $\hat {\pi } = 0$. This is a reassuring result and is congruent with the simulations in that for the preponderance of datasets the use of EM does not affect the results. However, when we move closer to a boundary, by for example removing 2 of the 3 cases with strong evidence of clonal relatedness (cases 36 and 48), the new approach corrects the estimation where the old approach was failing.

## Discussion

Our method provides a strategy for estimating, in a sample of cases with tumor pairs, the proportion of these cases that are clonally related, in addition to diagnostic probabilities for each case. As compared to other methods described in the introduction, the proposed model utilizes the information from a sample of patients, and includes all mutations observed in only one or in both tumors, in order to infer the probabilities of clonal relatedness. We now believe that an analysis of our proposed random-effects model should involve maximization of the likelihood using the EM algorithm rather than the one-step strategy based on conditioning on the latent clonality indicators that we had previously proposed. By doing so, we greatly reduce the chances that the estimator of the proportion of cases that are clonal will lead to an unsatisfactory boundary value. Of note, the increased performance comes at no cost regarding computation time. Our available R package *Clonality* [10] which includes the function to estimate the random-effects model, has been updated to adopt the EM strategy (version 1.32.0 and higher).

## Conclusion

The EM algorithm is a preferable approach for our clonality random-effects model. It is now the method implemented in our R package *Clonality*, making available an easy and fast way to estimate this model on a range of applications.

## Methods

The informative data *Y*_*j*_ for case *j* of *n* cases encompasses a set of indicators for the presence of shared or private mutations in the tumor pair at genetic loci denoted by *i*. [Private mutations are those that occur in one tumor but not in its pair.] The sets *A*_*j*_ and *B*_*j*_ contain the shared and private mutations respectively. We denote *G*_*j*_=*A*_*j*_∪*B*_*j*_. Each mutation *i* has a known probability of occurrence *p*_*i*_ in a tumor. Let *π* denote the proportion of clonal cases in the population, and *ξ*_*j*_ the clonality signal for case *j*. The clonality signal represents the relative period of tumor evolution in which mutations accrued in the originating clonal cell, and thus represents the anticipated proportion of mutations observed in a case that are matches. The term *C*_*j*_ represents the true clonal status of the tumor pair, taking the value 1 when the case is clonal and 0 when the case is independent. Note that *ξ*_*j*_=0 if *C*_*j*_=0. In clonal cases, we assume that − log(1−*ξ*_*j*_) has a lognormal density, with mean *μ* and standard-deviation *σ*. We use *g*(·) to denote density functions generically. As explained in Mauguen et al. [6], we previously used a conditional likelihood constructed in the following manner. Recognizing that

1 $$ {\begin{aligned} P\left(Y_{j} | \xi_{j}, C_{j} = 1 \right) = \prod_{i \in G_{j}} \!\left\{ \frac{\xi_{j} + (1-\xi_{j}) p_{i}}{\xi_{j} + (1-\xi_{j}) (2-p_{i})} \right\}^{I[i \in A_{j}]} \left\{ \frac{2(1-\xi_{j}) (1-p_{i})}{\xi_{j} + (1-\xi_{j}) (2-p_{i})} \right\}^{I[i \in B_{j}]} \end{aligned}}  $$

and

2 $$ P\left(Y_{j} | C_{j}=0 \right) = \prod_{i \in G_{j}} \left(\frac{p_{i}}{2-p_{i}} \right)^{I[i \in A_{j}]} \left\{ \frac{2 (1-p_{i})}{2-p_{i}} \right\}^{I[i \in B_{j}]}  $$

we elected to use case-specific likelihood contributions

$$L_{j}\left(\pi, \xi_{j} \right) = \pi P\left(Y_{j} | \xi_{j}, C_{j}=1 \right) + (1-\pi) P\left(Y_{j} | C_{j}=0 \right) $$

leading to

3 $$ L\left(\pi, \mu, \sigma \right) = \prod_{j=1}^{n} \int_{0}^{1} L_{j}\left(\pi, \xi_{j} \right) g(\xi_{j}) d\xi_{j}.  $$

This allowed us to perform the maximization to estimate simultaneously the parameters *π*,*μ*, and *σ* using a one-step Box constrained quasi-Newton algorithm. However, although in simulations the properties of this process appear to indicate low bias, we found that it is not uncommon, especially in small datasets or those where *π* is close to a boundary of 0 or 1, for the parameter *π* to have an Maximum Likelihood estimate of 0 or 1, rendering the diagnostic probabilities for all cases to be either 0 or 1. This problem is caused by the fact that the simplified conditional likelihood in (3) above does not fully recognize the influences of the case-specific mutational profiles *Y*_*j*_ on the case-specific clonality signals *ξ*_*j*_ and the individual levels of evidence regarding clonal relatedness *C*_*j*_. In short we used the parameter representing the overall probability of clonality *π* in (3) rather than the case-specific probabilities of clonality, *P*(*C*_*j*_=1|*ξ*_*j*_,*π*,*μ*,*σ*). To address this problem we employ a likelihood structure that permits a more specific use of these data from individual cases and have constructed a strategy involving the EM algorithm to estimate the parameters.

This approach recognizes the fact that the terms *C*_*j*_ and *ξ*_*j*_ are latent variables and that our goal is to maximize the likelihood that is not conditioned on these latent variables, i.e.

4 $$ L = \prod_{j=1}^{n} P\left(Y_{j} | \pi, \mu, \sigma \right).  $$

To perform the estimation we first recognize the following:

5 $$\begin{array}{*{20}l} P\left(Y_{j}, \xi_{j}, C_{j} | \pi, \mu, \sigma \right) = P\left(Y_{j} | \xi_{j}, C_{j} \right) \times g\left(\xi_{j}, C_{j} | \pi, \mu, \sigma \right) \end{array} $$

6 $$\begin{array}{*{20}l} = g\left(\xi_{j}, C_{j} | Y_{j}, \pi, \mu, \sigma \right) \!\times\! P\left(Y_{j} | \pi, \mu, \sigma \right). \end{array} $$

Note that the likelihood contribution of case j to (4) is a component of the right-hand side of (6). The EM algorithm permits us to instead maximize (iteratively) the expectation of the logarithm of this full likelihood, averaged over the latent variables conditioned on the data. That is, the expected likelihood is given by

7 $$ {\begin{aligned} E = \prod_{j=1}^{n} \int_{0}^{1} \log \left\{ P\left(Y_{j}, \xi_{j}, C_{j} | \pi, \mu, \sigma \right) \right\} g\left(\xi_{j}, C_{j} | Y_{j}, \tilde{\pi}, \tilde{\mu}, \tilde{\sigma} \right) d (\xi_{j}, C_{j}) \end{aligned}}  $$

where $\tilde {\pi }$, $\tilde {\mu }$, and $\tilde {\sigma }$ are the *current* estimates of the parameters. After choosing starting values for these parameters the expectation and maximization steps proceed iteratively until convergence. To calculate *E* we recognize that $P(Y_{j}, \xi _{j}, C_{j} | \tilde {\pi }, \tilde {\mu }, \tilde {\sigma })$ is obtained easily from the defined terms on the right-hand side of (5), represented by (1) and (2) and the parametric model used for the distribution of *ξ*_*j*_. Further, $g(\xi _{j}, C_{j} | Y_{j}, \tilde {\pi }, \tilde {\mu }, \tilde {\sigma })$ can be obtained from Bayes Theorem, i.e.

$${\begin{aligned} g\left(\xi_{j}, C_{j} | Y_{j}, \tilde{\pi}, \tilde{\mu}, \tilde{\sigma} \right) = \frac{g\left(\xi_{j}, C_{j} | \tilde{\pi}, \tilde{\mu}, \tilde{\sigma} \right) P\left(Y_{j} | \xi_{j}, C_{j} \right)} {\int_{0}^{1} g\left(\xi_{j}, C_{j} | \tilde{\pi}, \tilde{\mu}, \tilde{\sigma} \right) P\left(Y_{j} | \xi_{j}, C_{j} \right) d(\xi_{j}, C_{j})}. \end{aligned}} $$

## Abbreviations

EM: Expectation-maximization

## References

[1] Teixeira MR, Ribeiro FR, Torres L, Pandis N, Andersen JA, Lothe RA, Heim S (2004). Assessment of clonal relationships in ipsilateral and bilateral multiple breast carcinomas by comparative genomic hybridisation and hierarchical clustering analysis. Br J Cancer.

[2] Schultheis Anne M., Ng Charlotte K. Y., De Filippo Maria R., Piscuoglio Salvatore, Macedo Gabriel S., Gatius Sonia, Perez Mies Belen, Soslow Robert A., Lim Raymond S., Viale Agnes, Huberman Kety H., Palacios Jose C., Reis-Filho Jorge S., Matias-Guiu Xavier, Weigelt Britta (2015). Massively Parallel Sequencing-Based Clonality Analysis of Synchronous Endometrioid Endometrial and Ovarian Carcinomas. Journal of the National Cancer Institute.

[3] Perea J, García JL, Corchete L, Lumbreras E, Arriba M, Rueda D, Tapial S, Pérez J, Vieiro V, Rodríguez Y, Brandáriz L, García-Arranz M, García-Olmo D, Goel A, Urioste M, Sarmiento RG (2019). Redefining synchronous colorectal cancers based on tumor clonality. Int J Cancer.

[4] Cereda M, Gambardella G, Benedetti L, Iannelli F, Patel D, Basso G, Guerra RF, Mourikis TP, Puccio I, Sinha S, Laghi L, Spencer J, Rodriguez-Justo M, Ciccarelli FD (2016). Patients with genetically heterogeneous synchronous colorectal cancer carry rare damaging germline mutations in immune-related genes. Nat Commun.

[5] Bao L, Messer K, Schwab R, Harismendy O, Pu M, Crain B, Yost S, Frazer KA, Rana B, Hasteh F, Wallace A, Parker BA (2015). Mutational Profiling Can Establish Clonal or Independent Origin in Synchronous Bilateral Breast and Other Tumors. PLoS ONE.

[6] Mauguen A, Seshan VE, Ostrovnaya I, Begg CB (2018). Estimating the probability of clonal relatedness of pairs of tumors in cancer patients. Biometrics.

[7] Ostrovnaya I, Seshan VE, Olshen AB, Begg CB (2011). Clonality: an R package for testing clonal relatedness of two tumors from the same patient based on their genomic profiles. Bioinformatics.

[8] Begg CB, Ostrovnaya I, Geyer FC, Papanastasiou AD, Ng CKY, Sakr RA, Bernstein JL, Burke KA, King TA, Piscuoglio S, Mauguen A, Orlow I, Weigelt B, Seshan VE, Morrow M, Reis-Filho JS (2018). Contralateral breast cancers: Independent cancers or metastases?. Int J Cancer.

[9] Ellrott K, Bailey MH, Saksena G (2018). Scalable Open Science Approach for Mutation Calling of Tumor Exomes Using Multiple Genomic Pipelines. Cell Syst.

[10] Ostrovnaya I. Clonality: Clonality testing. 2019. R package version 1.32.0.

